# Supplementary material for: Selective ligand removal to improve accessibility of active sites in hierarchical MOFs for heterogeneous photocatalysis
Source: Nat Commun. 2022 Jan 12;13:282. doi: 10.1038/s41467-021-27775-7 (PMC8755752; doi:10.1038/s41467-021-27775-7)
Supplement: Supplementary file 1 — Supplementary Information [file 41467_2021_27775_MOESM1_ESM.pdf]

Supplementary Information

Selective ligand removal to improve accessibility of active sites in hierarchical MOFs for heterogeneous photocatalysis

Naghdi et al.

Supplementary Figures

Supplementary Tables

Supplementary Methods

Supplementary Notes | Synthesis

Supplementary Notes | DFT Calculations

Supplementary References

Supplementary Figures

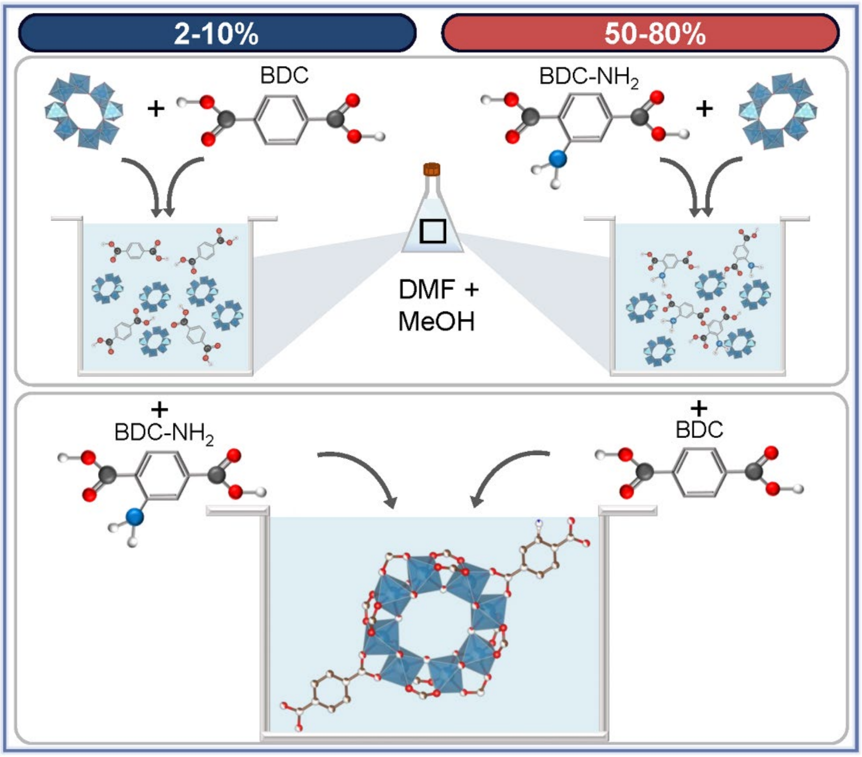

Supplementary Fig. 1 | Schematic representation of the two synthesis routes. Schematic representation of the two synthesis routes. The samples with low BDC-NH_2 contents are produced by first adding BDC to a TTIP solution to obtain MIL-125-Ti nuclei to which subsequently the corresponding amounts of BDC-NH_2 were added. The order of the addition of the respective ligands was reversed for the samples with high BDC-NH_2 contents.

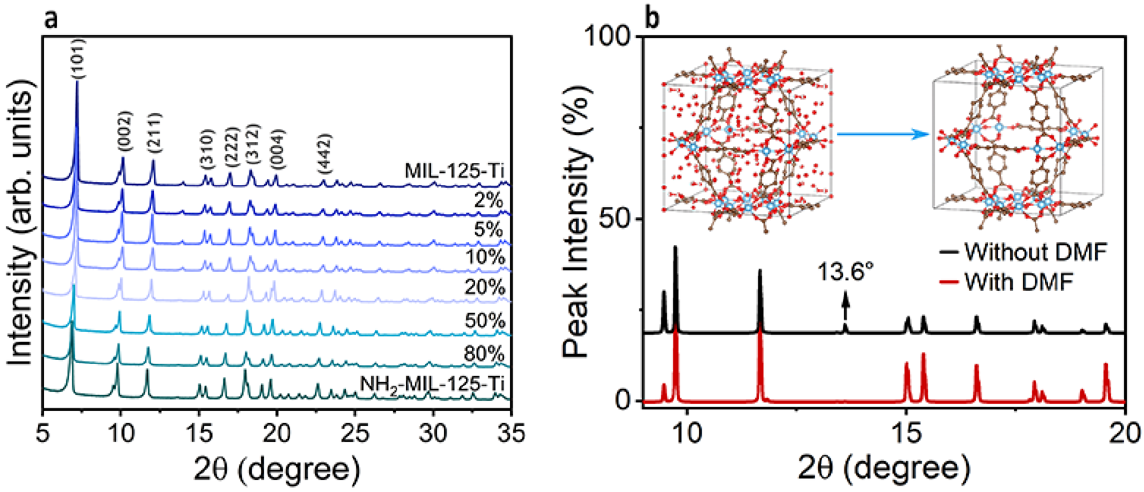

Supplementary Fig. 2 | Structural characteristics of as-prepared mixed-ligand MOFs. **(a)** XRD of the as-prepared mixed-ligand MOFs with different BDC-NH_2 ratios of 0% (MIL), 2%, 5%, 10%, 50%, 80% and 100% (NH_2-MIL). All samples are highly crystalline and exhibit the typical MIL structure of the single-ligand MOFs (0% and 100%)[1]. Note the absence of minority phases and impurities. (**b**). Simulated PXRD patterns of MIL-125-Ti with and without solvent molecules (DMF) inside the pores. The key feature here is the prominent peak at 13.6°, which is only visible for the simulated pattern in absence of DMF which is typically incorporated within the micropores upon synthesis. Note that the peak intensity correlates inversely to the amount of the solvent. Hence, it grows in intensity when the DMF gets removed by evaporation (e.g., upon heat treatment, see Supplementary Fig. 5). A small 13.6° peak is also present in the as-prepared samples and its intensity seems to depend on the BDC-NH_2 content. The polar amino groups of the ligand may facilitate the incorporation of DMF molecules, consequently decreasing the peak intensity with increasing BDC-NH_2 content. However, the rather abrupt change in intensity between the 10% and 50% sample may suggest that the order of ligand addition before crystallization has an effect on the DMF content as well. Presumably, the presence of BDC-NH_2 in all mixed-ligand nuclei of route 2 attracts more DMF molecules, compared with the single-ligand nuclei of route 1, where DMF is confined to the pure BDC-NH_2 nuclei (max 10%).

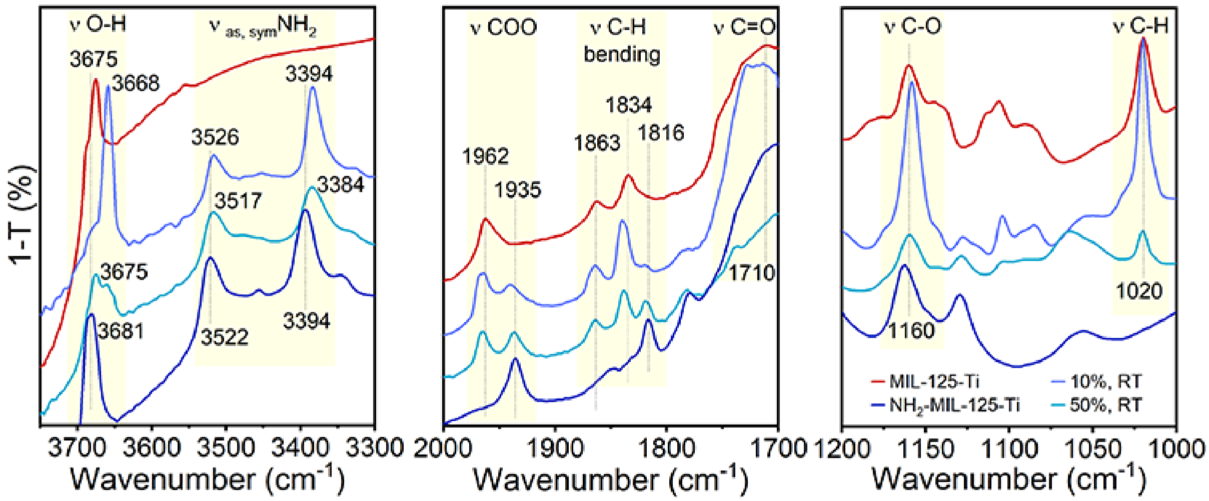

Supplementary Fig. 3 | Characterization of as-prepared mixed-ligand MOFs. DRIFTS spectra for MIL, NH_2-MIL, 10%NH_2-MIL, and 50%NH_2-MIL collected at room temperature. The shaded regions mark the crucial vibrational features that are discussed in the main manuscript. Note that the features in the mixed-ligand MOFs (e.g., COO^- at 1930-1960 cm^{-1} and CH at 1810-1860 cm^{-1}) are generally superimposed from those in the single-ligand MOFs. The region of the OH vibrations associated with the SBU is more complex and discussed in the main text.

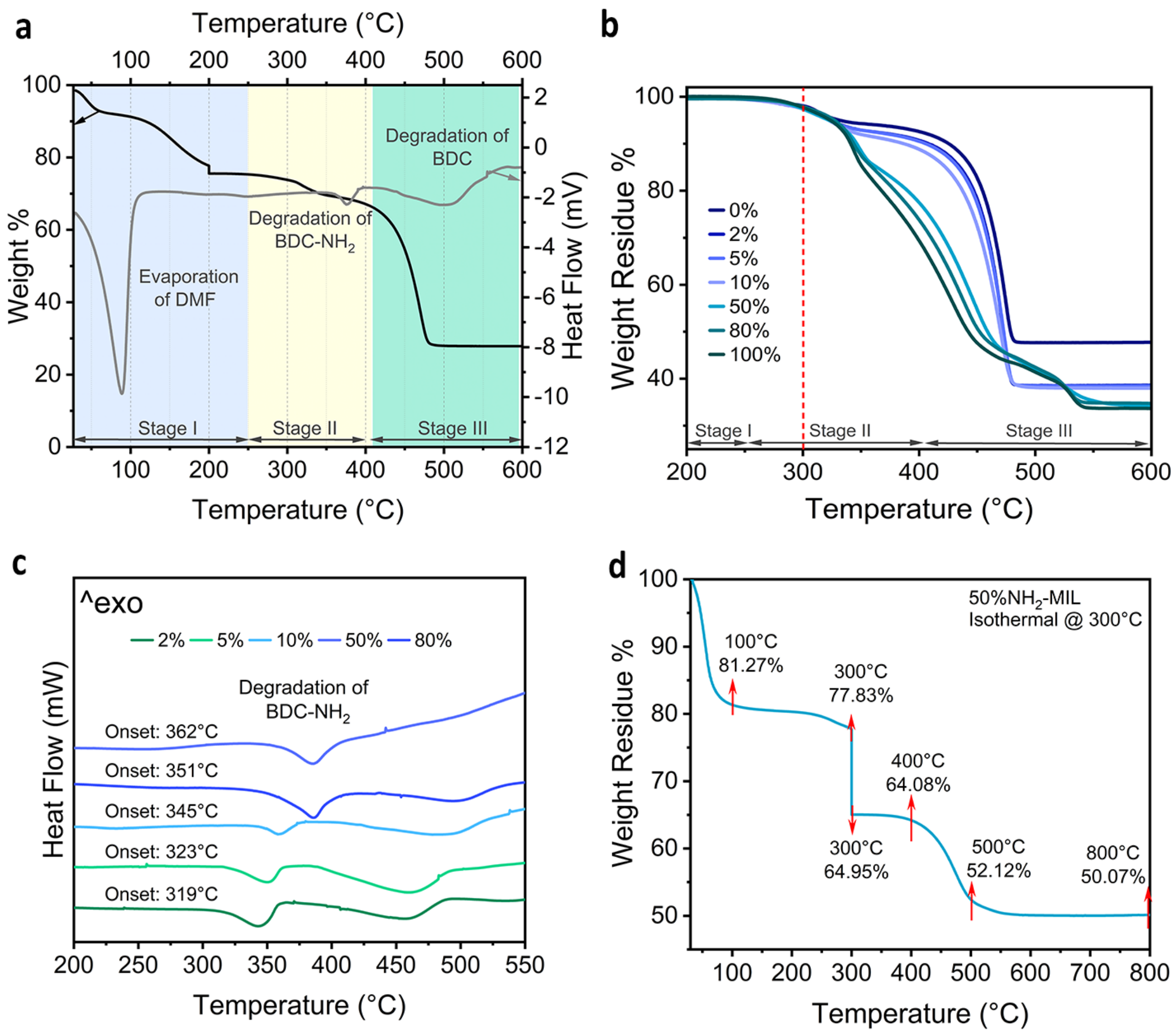

Supplementary Fig. 4 | Characterization of mixed-ligand MOFs upon heat treatment. (**a**) TGA and DSC curves for 10%NH_2-MIL, using a ramp rate of 10 °C min^{-1} and a low rate of 20 mL min^{-1} in air. Stage I indicates the removal of solvents, (DMF); Stage II marks the selective removal of BDC-NH_2; stage III shows the degradation of BDC. (**b**) TGA curves for the single-ligand and mixed-ligand MOFs. All samples experience a weight loss at 300-350 °C with its extent increasing with increasing BDC-NH_2 content. (**c**) DSC of mixed-ligand MOFs showing the onset temperatures of the endothermic peak representing BDC-NH_2 degradation, which increased slightly for 2%NH_2-MIL to 80%NH_2-MIL. (**d**) TGA of mixed-ligand 50%NH_2-MIL that was heated to 300 °C and kept for 20 hours before continuing ramping. During the isothermal heating, the weight was reduced by about 15%, corresponding to BDC-NH_2. The subsequent weight-loss beyond 400 °C also amasses to 15% and corresponds to the removal of BDC. This separated 2-step process at different temperatures with equal weight losses is expected from 50%NH_2-MIL. It confirms the high ligand removal selectivity of NH_2-BDC and documents that all NH_2-BDC removed at 300 °C.

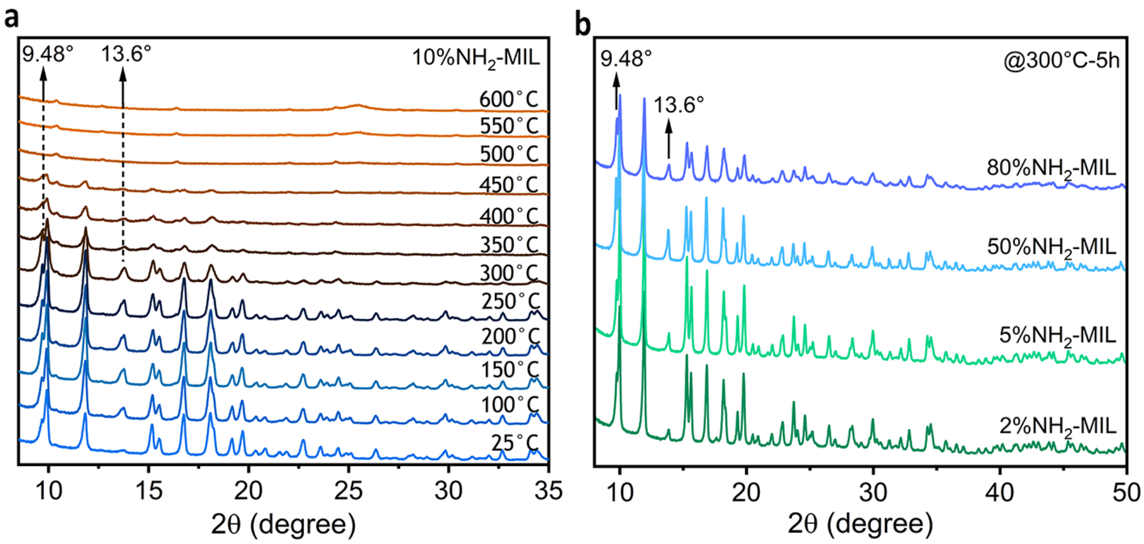

Supplementary Fig. 5 | Crystal structure of mixed-ligand MOFs at different temperature (ex situ) and during ramping. (**a**) XRD pattern of 10%NH_2-MIL from RT to 600 °C. (**b**) XRD pattern of 2%, 5%, 50% and 80%NH_2-MIL heated at 300 ° for 5 h. All samples show a small diffraction peak at 2θ of 13.6° as its intensity concerning the prominent MOF peaks decreases with the increase of BDC-NH_2 content. The simulated XRD pattern in the Supplementary Figure 2b also explains that this peak is related to the solvent molecules (DMF) incorporated within the micropores during synthesis. The peak intensity is inversely correlated to the amount of the residual solvent. Therefore, an increase of BCD-NH_2 content facilitates the incorporation of solvent molecules, potentially due to the additional presence of the polar amino group.

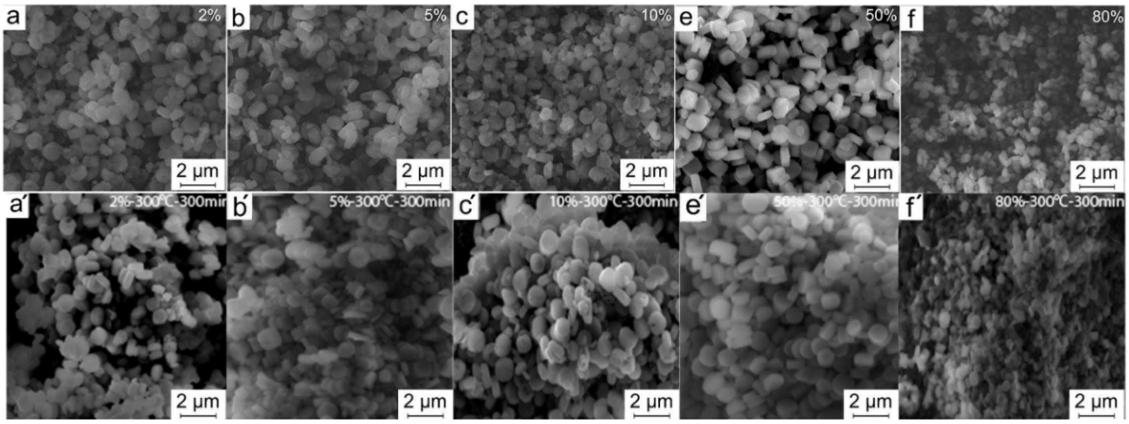

Supplementary Fig. 6 | Morphology and particle size. (**a-f**) SEM images of the mixed-ligand MOFs for the as-prepared samples (2%-80%NH_2-MIL). (**a'-f'**) Heated-treated samples at 300 °C for 5 h. All samples show particles of similar circulate plate-like shape. The increase of the BDC-NH_2 ratio in the samples led to a decrease of the particle size, as quantified below. Note that the heat treatment has changed neither morphology nor size significantly.

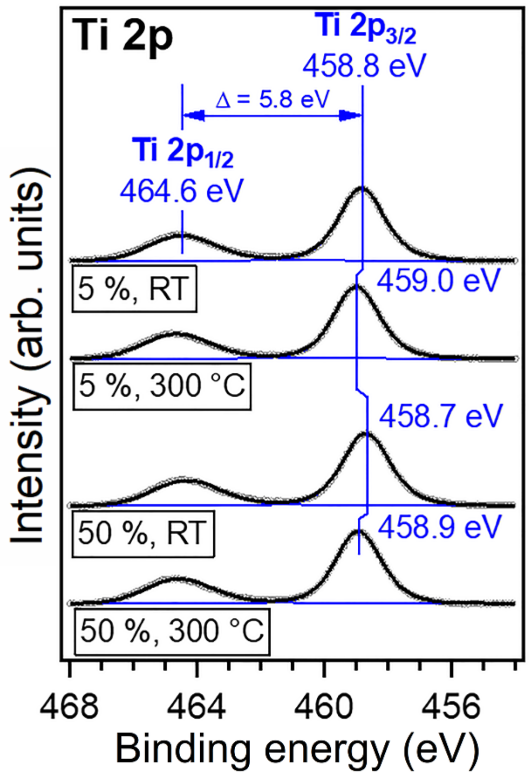

Supplementary Fig. 7 | Presence of TiO_2 nanoparticles. XPS Ti-*2p* spectra for the 5%NH_2-MIL and the 50%NH_2-MIL, both as-prepared and heated at 300 °C for 5 h. Note that the binding energies of the peaks are almost the same in all cases, which indicates that the MOF framework remains intact. It also means that no TiO_2 particles are present, which would exhibit about 2 eV lower binding energies.

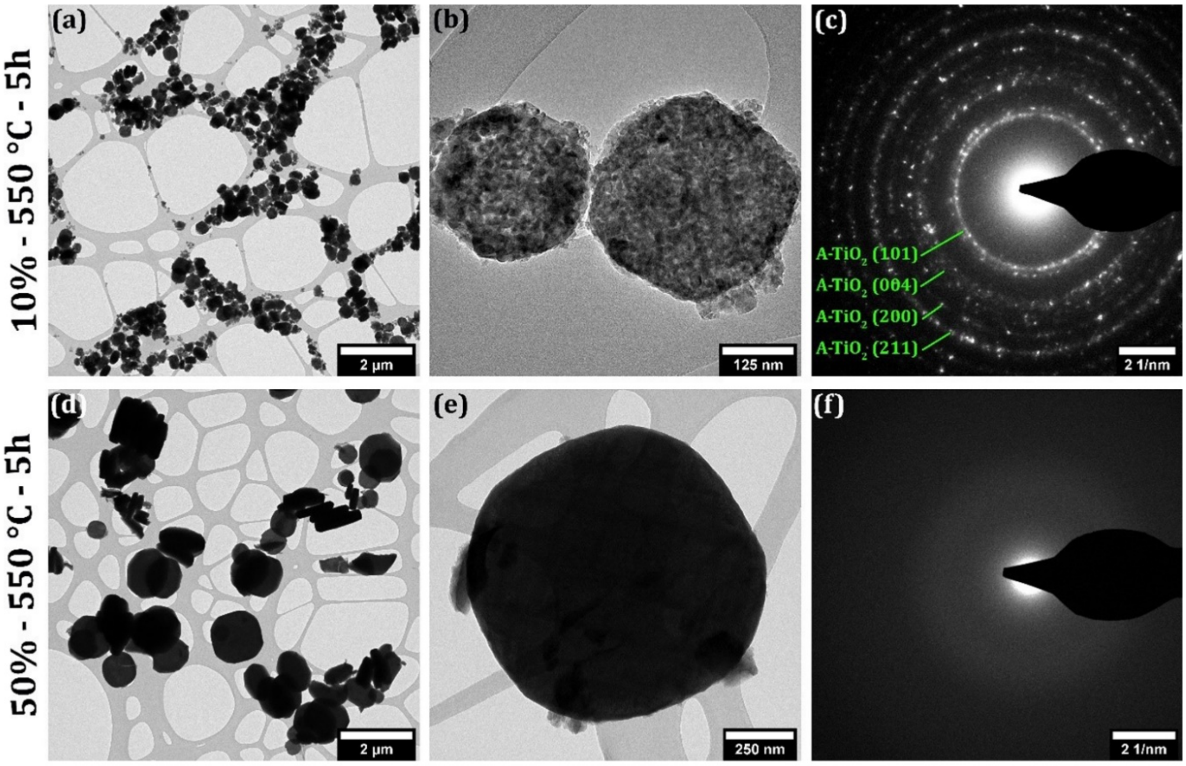

Supplementary Fig. 8 | Presence of TiO_2 nanoparticles - TEM and SAED. BF-TEM overview images of the 10%NH_2-MIL (**a**) and 50%NH_2-MIL (**d**) heated at 550 °C for 5 h. Mixed-ligand MOFs with respective individual MOF particles shown in (**b**) and (**e**), respectively. The SAED pattern recorded on the 10%NH_2-MIL heated at 550 °C for 5 h (**c**) confirms the complete conversion of MOF particles to polycrystalline anatase TiO_2. The diffraction pattern in (c) was indexed with the help of the pdf file number 04-011-0664[2] from the PDF4+ 2021 database and found to correspond to anatase TiO_2. The electron diffraction pattern recorded on the 50%NH_2-MIL heated at 550 °C for 5 h (**f**) did not show any reflections suggesting the possible complete damage of the 50% mixed-ligand MOF particle on heat treatment to 550 °C.

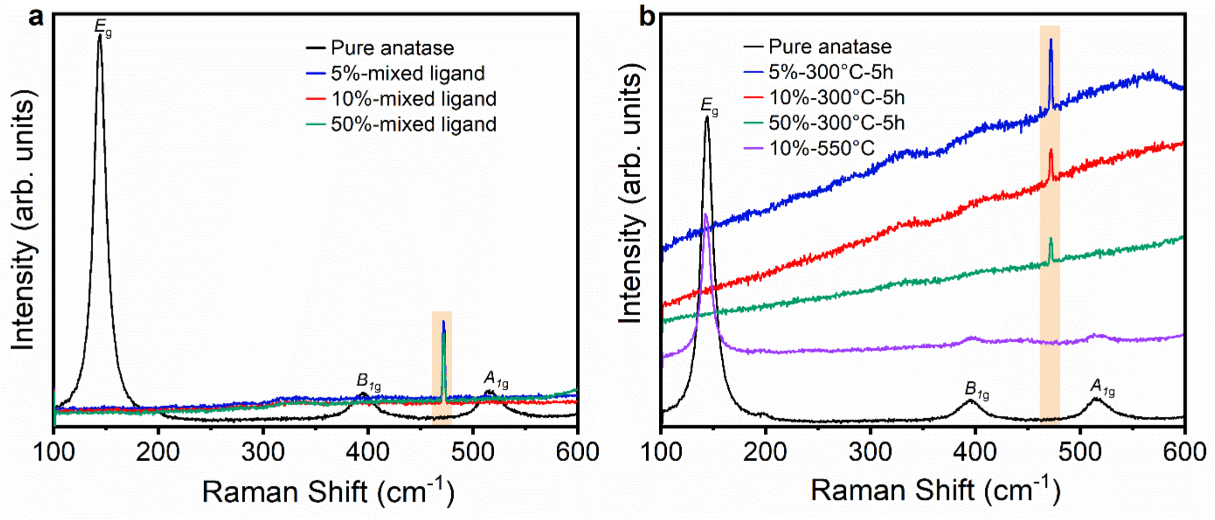

Supplementary Fig. 9 | Presence of TiO_2 nanoparticles. Raman spectra obtained from 5%, 10%, and 50%NH_2-MIL mixed-ligand MOFs in their as-prepared state (**a**) and after being heat-treated at 300 °C for 5 h (**b**). Both graphs also show a spectrum of anatase TiO_2 nanoparticles[3] as a reference. The peak at ~ 472 cm^{-1} marks the fingerprint feature for MIL-based MOFs, in accordance with literature[4], and is visible in all MOF samples. The peak is preserved upon heating to 300 °C, which indicates that the MOF structure has not been altered, which is consistent with XRD and SAED/TEM data. The relative decrease in intensity is attributed to the increase in background noise related to fluorescence. Importantly, there are no signs of any minority phases, such as TiO_2, for neither as-prepared nor heat-treated samples. The observed Raman bands at 144.57, 395.25, 515.52 cm^{-1} correspond to anatase's E_g, B_{1g}, A_{1g} modes[5].

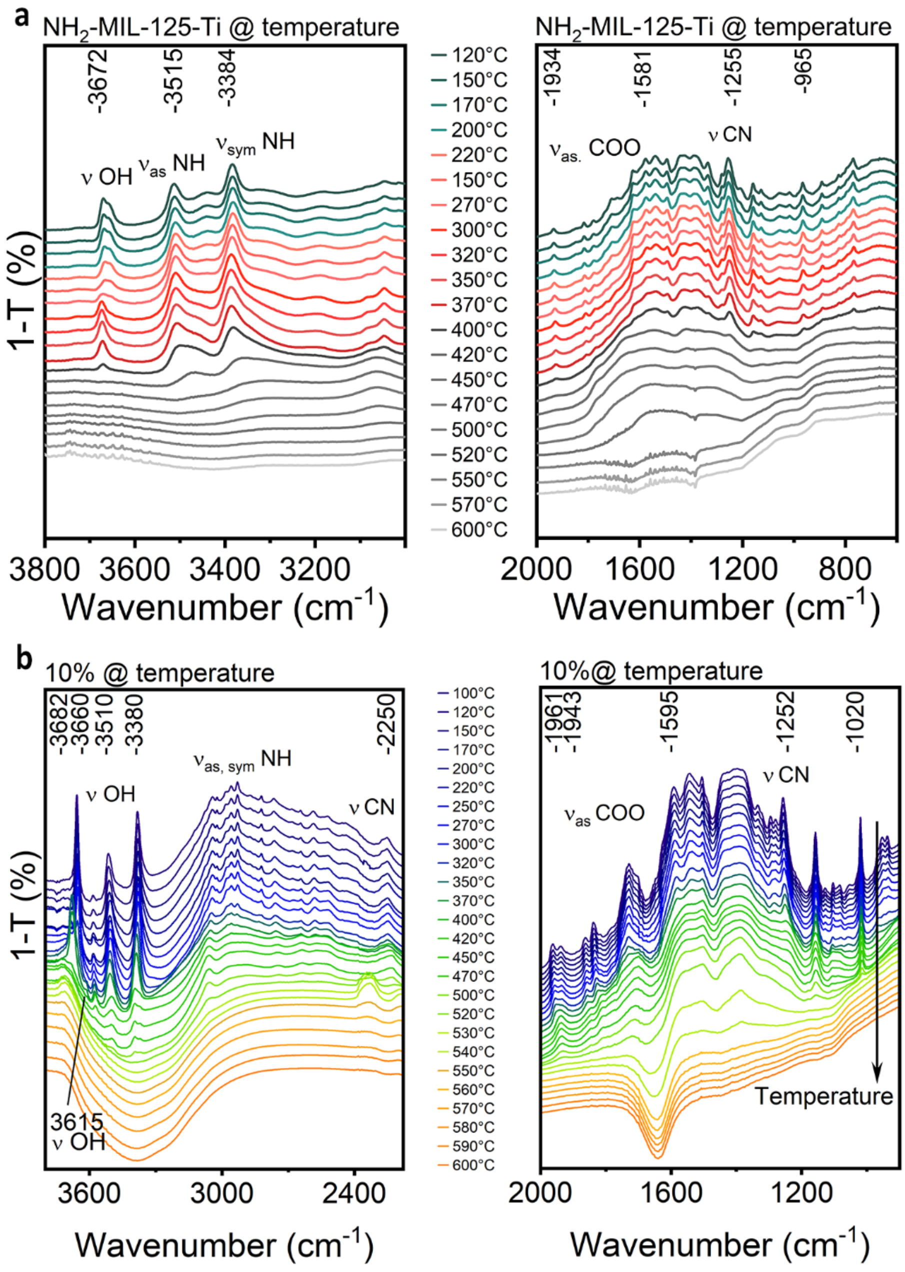

Supplementary Fig. 10 | IR Spectroscopic information on ligand removal upon ramping. The figures show DRIFS data on single-ligand NH_2-MIL MOFs (**a**) and 10%NH_2-MIL mixed-ligand MOFs (**b**). Detailed description of the changes in IR bands upon heat treatment can be seen in the main text.

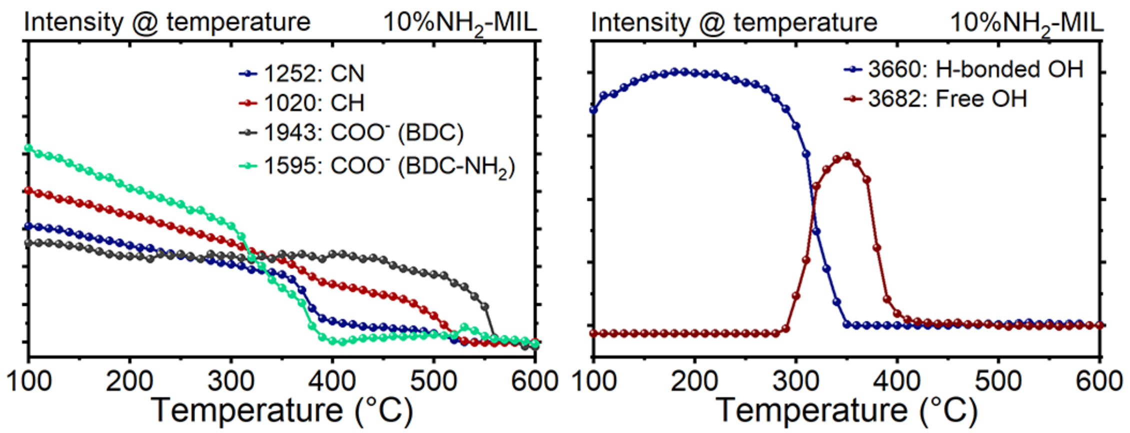

Supplementary Fig. 11 | Evolution of characteristic IR bands, upon temperature. Characteristic IR bands from 100 °C to 600 °C for 10%NH_2-MIL. Note that the COO^- band of BDC-NH_2 decreases in two steps between 300 °C and 400 °C, while the corresponding COO^- band of BDC remains unchanged.

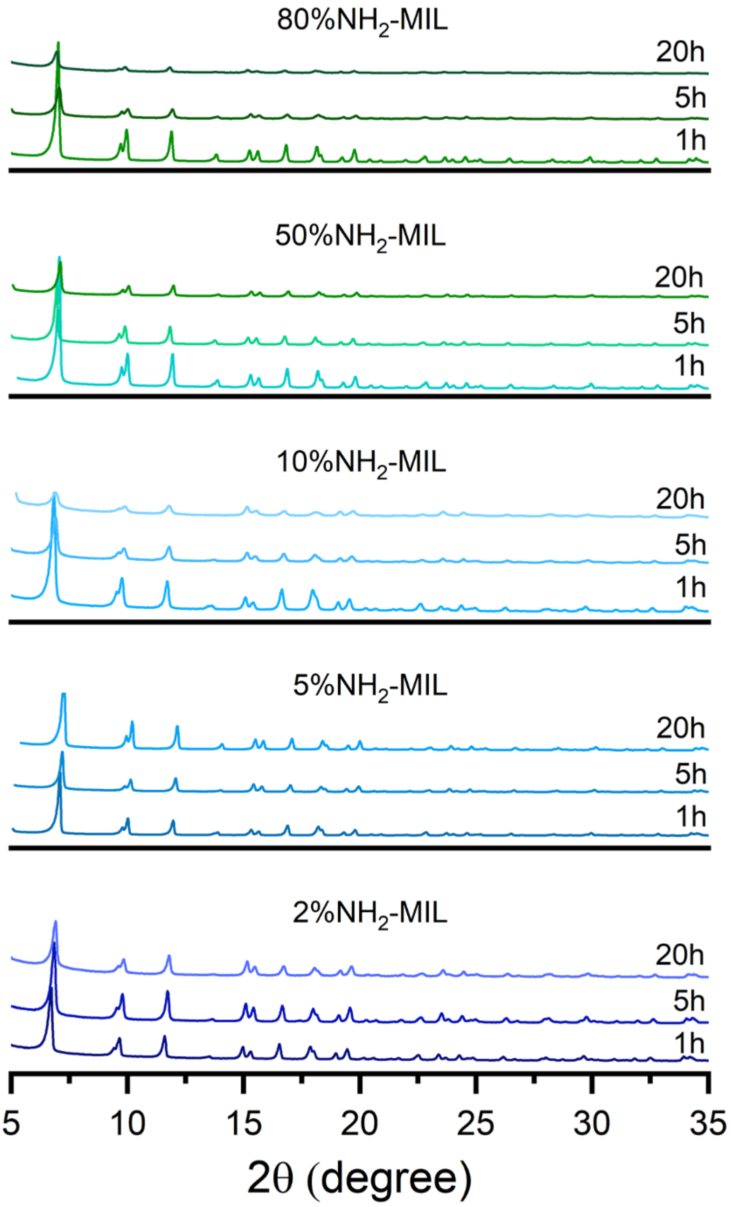

Supplementary Fig. 12 | Crystal structure of Mixed-ligand MOFs upon isothermal heating. ex situ XRD patterns for heat-treated MOFs at 300 °C for 1, 5 and 20 h. All patterns show sharp diffraction peaks indicate high crystallinity and the absence of impurity compounds or secondary phases. The ligand content did not significantly change the unit cell of the MOFs, which is expected considering that the mixed ligand MOFs consist of ligands with common coordination directionality[6].

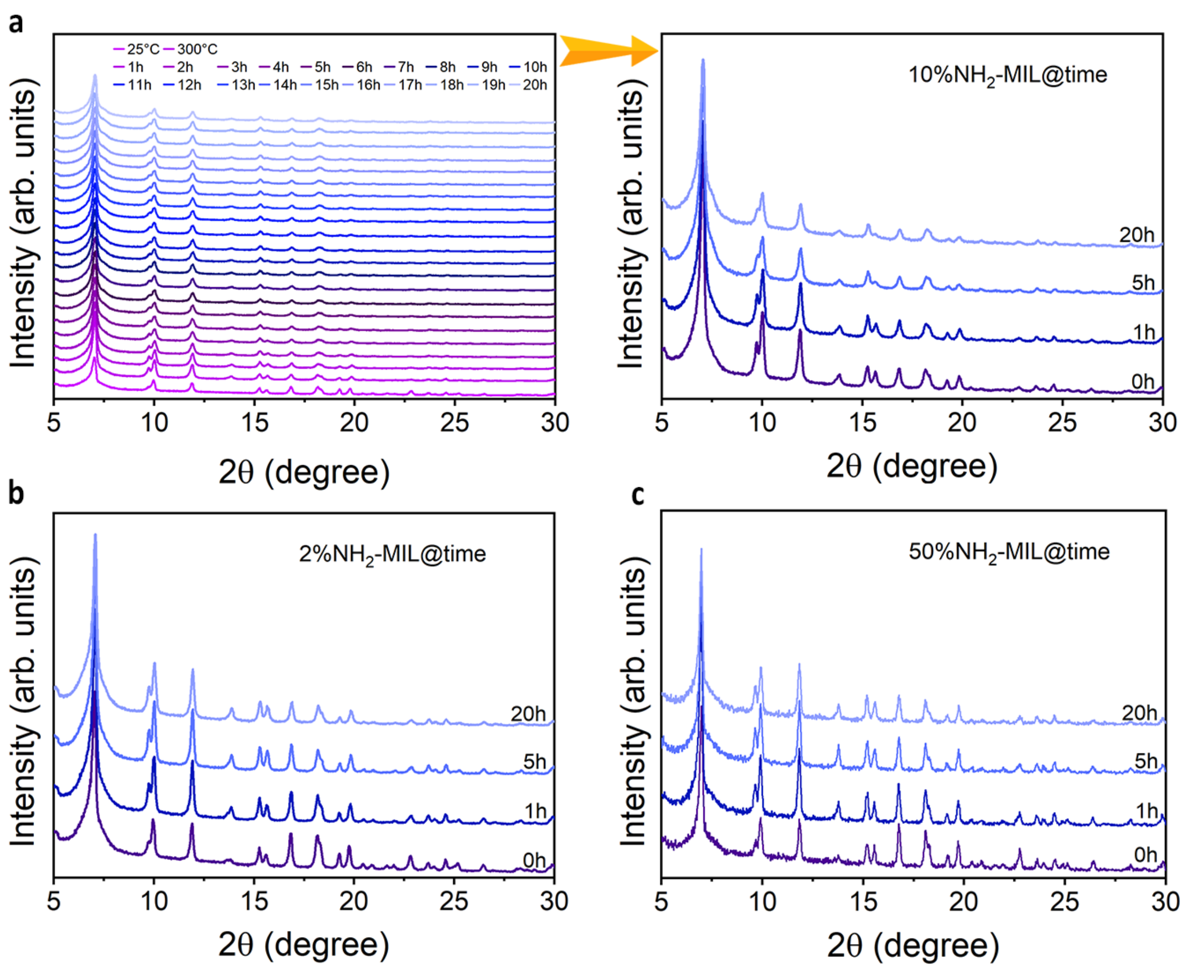

Supplementary Fig. 13 | in situ characterization of mixed-ligand MOFs upon heating. in situ XRD patterns of 10%NH_2-MIL (**a**) 2%NH_2-MIL (**b**) 50%NH_2-MIL (**c**) taken during heat treatment in air (0.5 mL min^{-1}) up to 20 hours (5 °C min^{-1}).

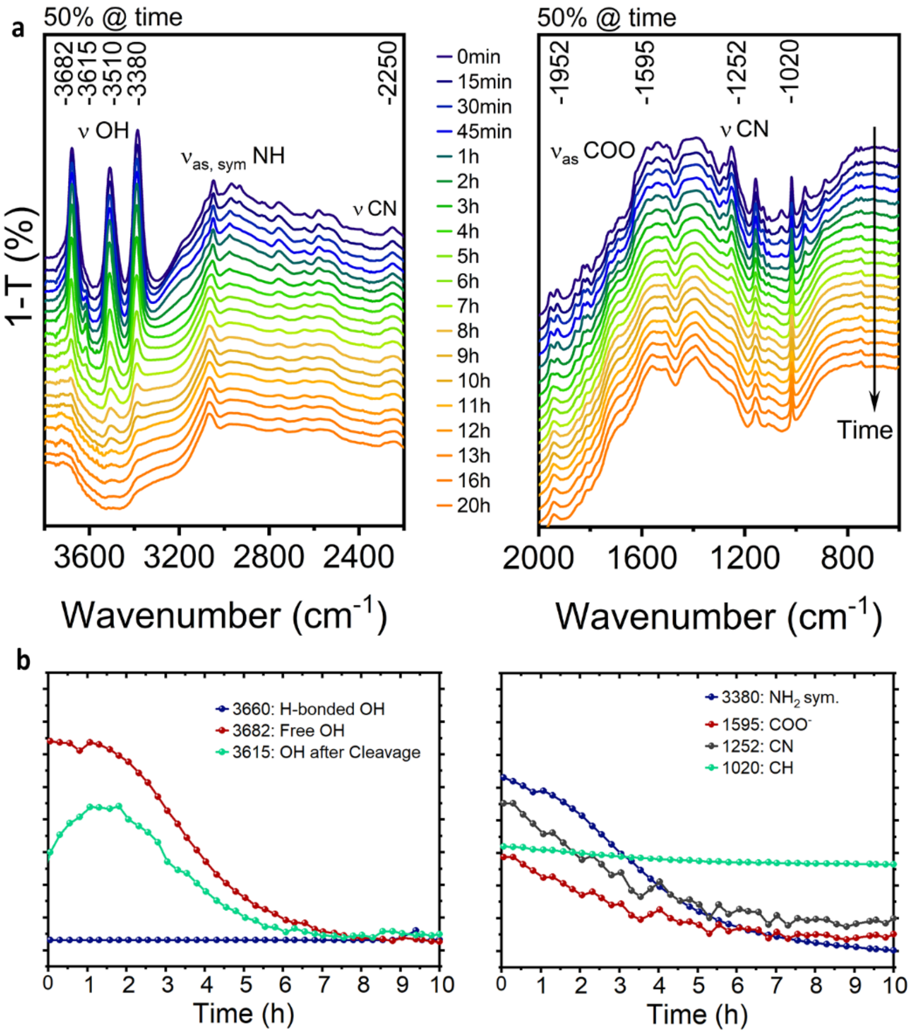

Supplementary Fig. 14 | IR Spectroscopic information on ligand removal upon isothermal heating. (**a**) in situ DRIFTS study of 50%NH_2-MIL MOFs during isothermal heating up to 20 h. The spectra highlight the key IR-active features referred to in the main text. (**b**) Isothermal evolution plots of the key IR bands. Similar to the 10%NH_2-MIL (main text Fig. 5), the isothermal DRIFTS spectra show a two-step process of BDC-NH_2 degradation at 300 °C.

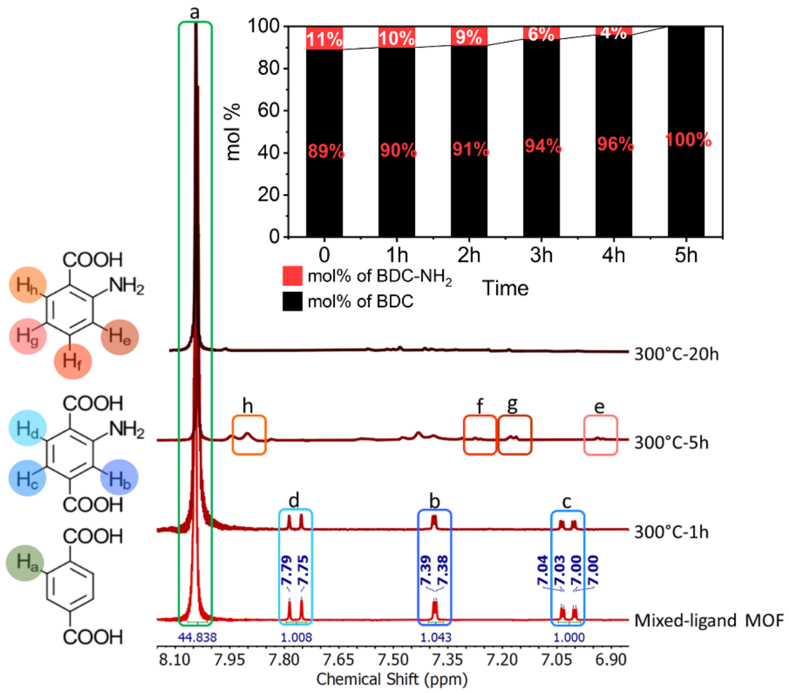

Supplementary Fig. 15 | ^{1}HNMR-spectroscopic information on ligand removal upon isothermal heating. ^{1}HNMR of the mixed-ligand MOFs and heat-treated MOFs for 1, 5, 20 h and the calculated ratio of $BDC-NH_2$ mol% in 10%NH_2-MIL sample upon the time of heat treatment from 1 h to 5 h. The spectrum of the mixed-ligand MOF shows the typical features expected from the BDC and $BDC-NH_2$ ligands[7]. Heat treatment has affected the spectra noticeably: while the $BDC-NH_2$-related chemical shifts are still clearly visible after 1 h, the spectrum after 5 h shows that almost all the ligands have converted into aminobenzoic acid species (7.92, 7.25, 7.15, 6.91 ppm). The spectrum of the sample heated for 20 h shows no significant peaks anymore (beside the BDC ligand), confirming the complete removal of $BDC-NH_2$.

Mixed-ligand MOF: *^1H NMR (250 MHz, DMSO-d_6) δ 8.04 (s, 4H), 7.77 (d, J = 8.3 Hz, 1H), 7.39 (d, J = 1.6 Hz, 1H), 7.02 (dd, J = 8.3, 1.7 Hz, 1H).* Heat-treated MOF at 300 °C for 1 h: *^1H NMR (250 MHz, DMSO-d_6) δ 8.04 (s, 4H), 7.77 (d, J = 8.3 Hz, 1H), 7.39 (d, J = 1.7 Hz, 1H), 7.02 (dd, J = 8.3, 1.7 Hz, 1H).* Heat-treated MOF at 300 °C for 5 h: *^1H NMR (250 MHz, DMSO-d_6) δ 8.04 (s, 4H), 7.92 (d, J = 7.5 Hz, 1H), 7.49 – 7.35 (m, 1H), 7.25 (dd, J = 7.5, 1.6 Hz, 1H), 7.15 (dd, J = 7.5, 1.5 Hz, 1H), 6.91 (dd, J = 7.5, 1.6 Hz, 1H).* Heat-treated MOF at 300 °C for 20 h: *^1H NMR (250 MHz, DMSO-d_6) δ 8.04 (s, 4H), 7.96 (d, J = 2.7 Hz, 1H), 7.59 – 7.36 (m, 1H), 7.23 (d, J = 1.5 Hz, 1H), 7.19 (d, J = 1.5 Hz, 1H).*

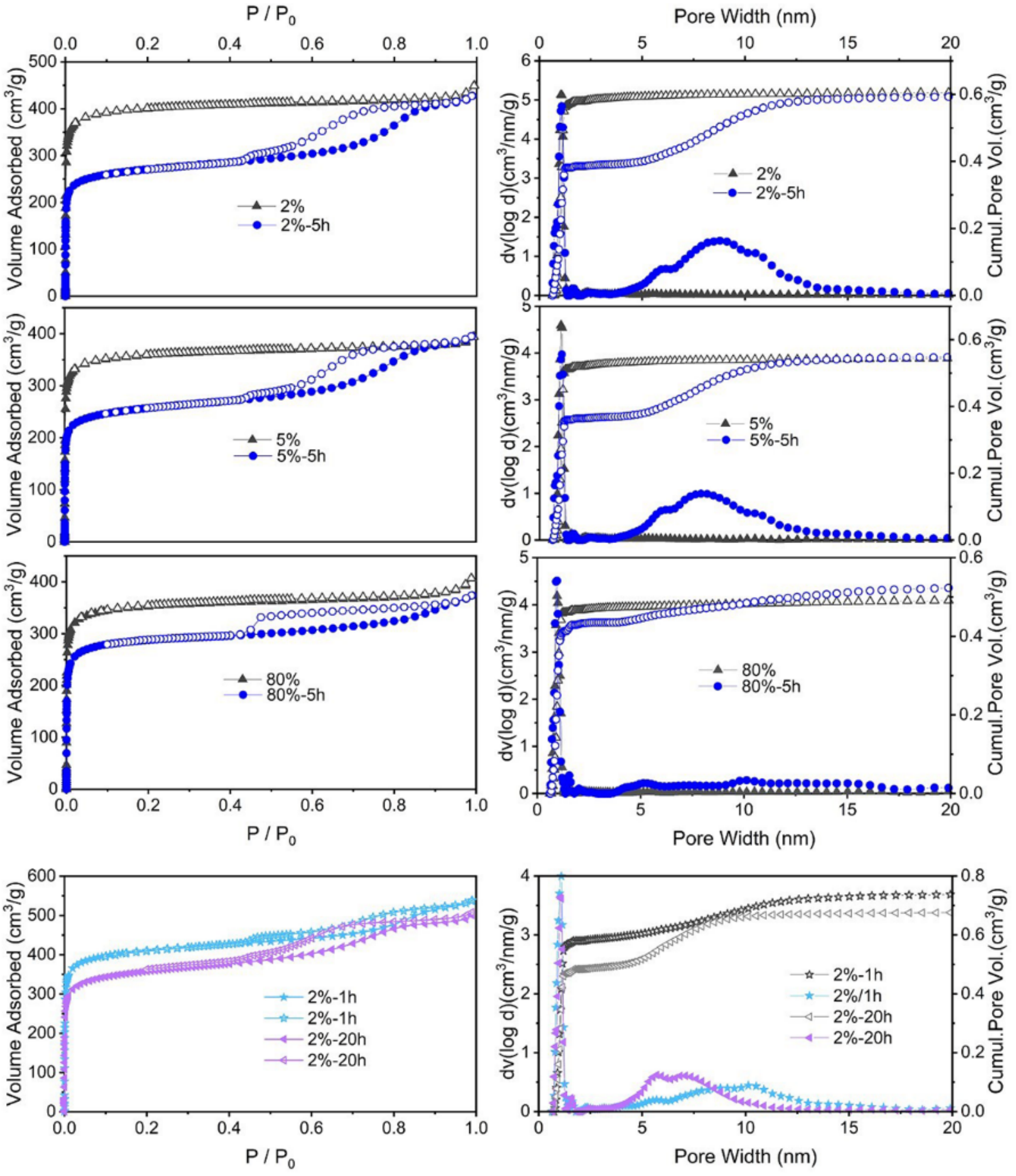

Supplementary Fig. 16 | Ar physisorption isotherms and pore size distribution. Comparison of Ar physisorption studies of as-prepared 2%, 5%, and 80%NH_2-MIL mixed-ligand and heat-treated samples at 300 °C for 1, 5 and 20 h. Samples with 2%, and 5%NH_2-MIL are same as 10%NH_2-MIL (Fig. 6a,b), which can be attributed to the similar synthesis route. In comparison, the samples with 80%NH_2-MIL is identical to the sample with 50%NH_2-MIL (Fig. 6d,e).

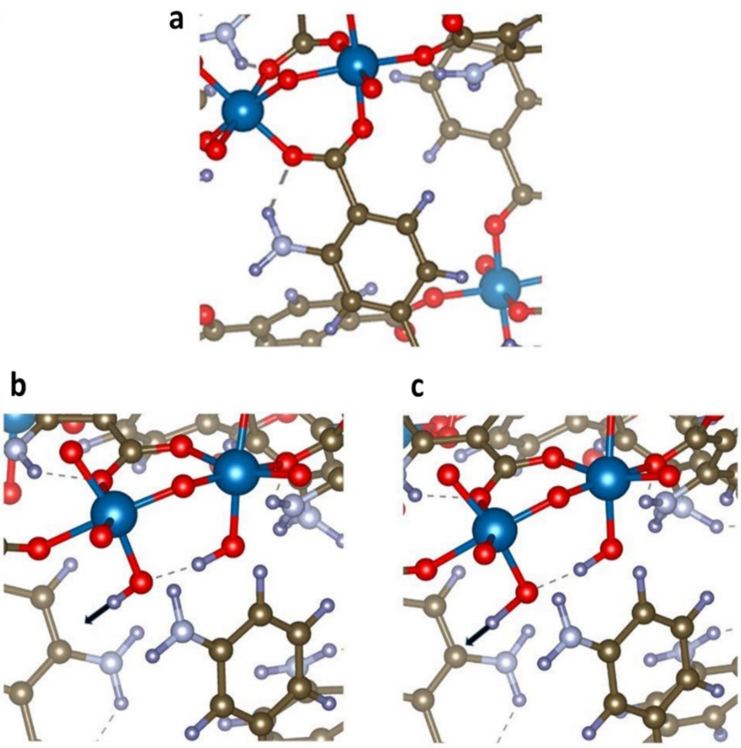

Supplementary Fig. 18 | Simulated structure of ligand removal. (**a**) Simulated structure of NH_2-MIL before Cleavage. (**b,c**) Vibration mode of newly created hydroxyl groups on Ti as a result of COO-Ti bond cleavage.

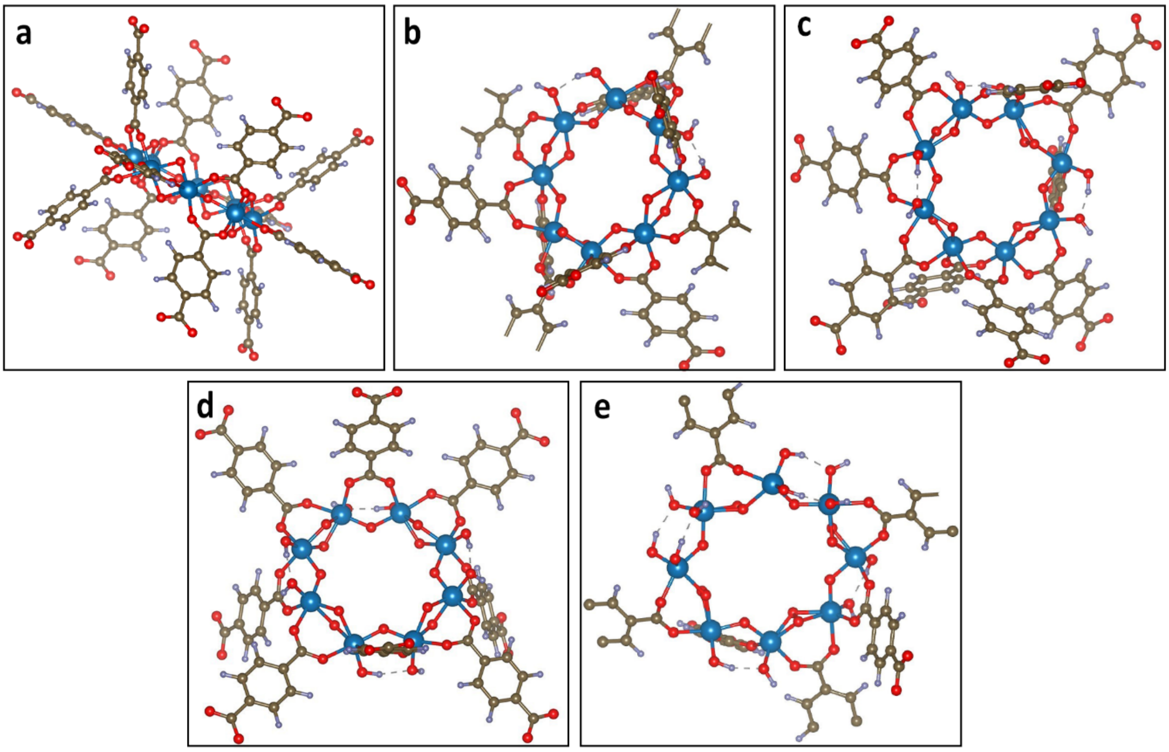

Supplementary Fig. 19 | Schematic representations of the MIL frameworks after ligand removal. (**a**) MIL framework with 0/12 ligands removed, Etot = -1680.8546 eV. (**b**) MIL framework with 2/12 ligands removed, Etot = -1527.3046 eV. (**c**) MIL framework with 3/12 ligands removed, Etot = -1453.3950 eV. (**d**) MIL framework with 4/12 ligands removed, Etot = -1374.4798 eV. (**e**) MIL framework with 6/12 ligands removed, Etot = -1234.4799 eV.

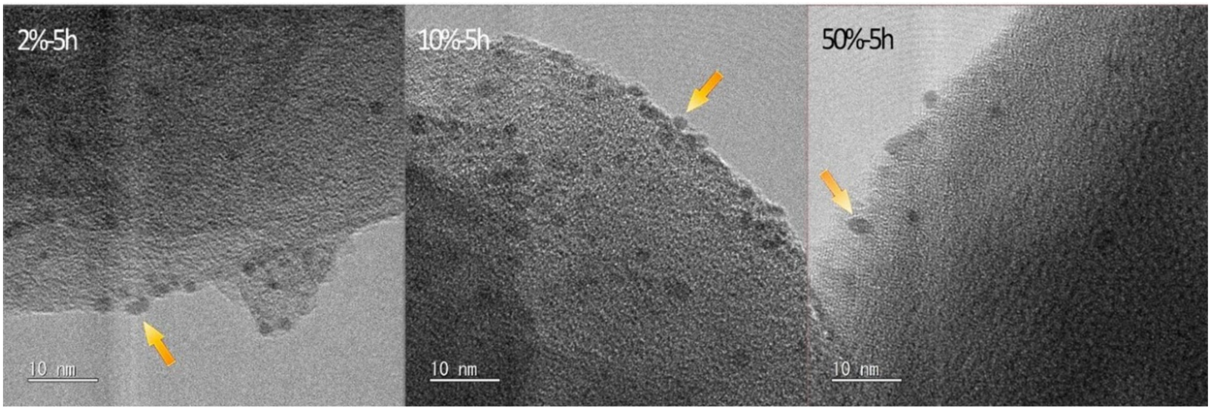

Supplementary Fig. 19 | Stability of the photocatalyst. TEM images of the 2%, 10%, and 50%NH_2-MIL samples heated at 300 °C for 5 h loaded with Pt co-catalyst, taken after a typical HER experiment. The images show small Pt particles immobilized on the MOFs particles with the typical average size of about 2 nm[8]. Neither the average size, nor their dispersion on the MOF particle surface has changed significantly upon HER reaction.

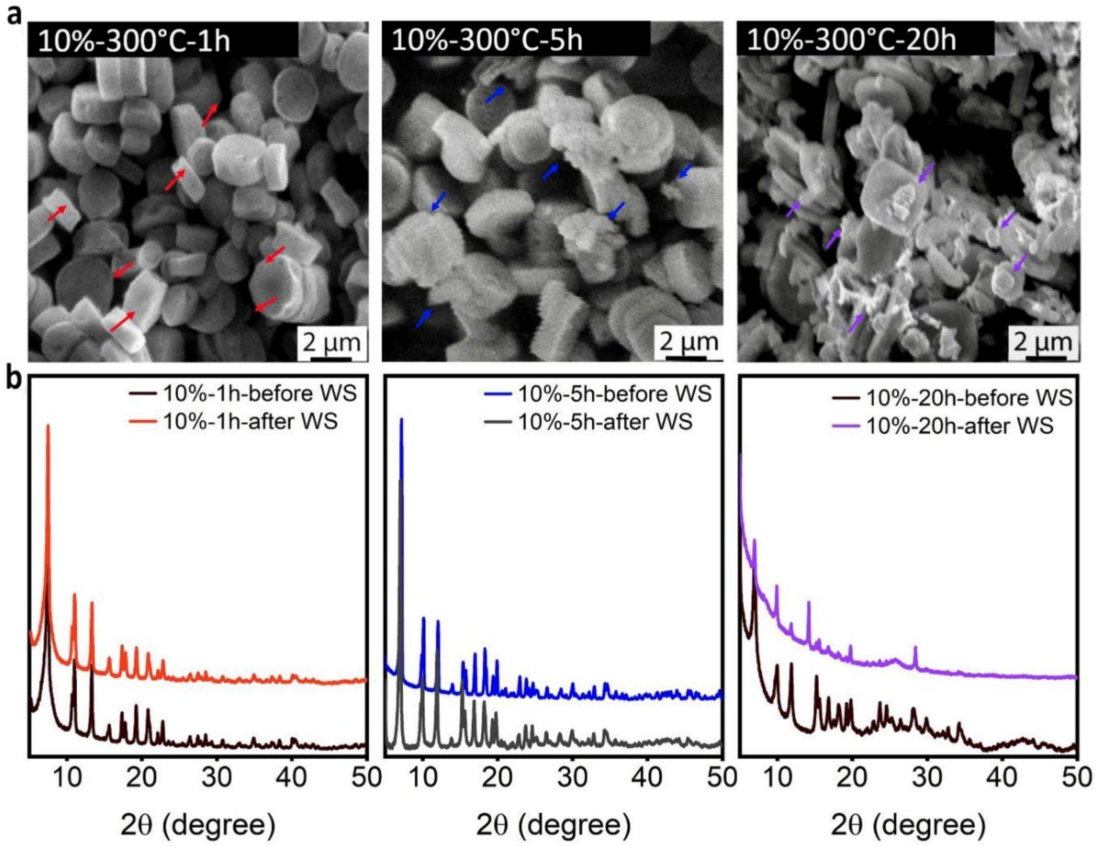

Supplementary Fig. 20 | Stability of the photocatalyst. SEM images (**a**) and corresponding XRD pattern (**b**) of the 10%NH_2-MIL mixed-ligand and heat-treated sample at 300 °C for 1, 5, 20 h – before and after photocatalytic HER experiments. Note that the samples heated for 1 h and 5 h exhibit no changes in crystal structure, nor in morphology and size of the MOF particles. Only the sample treated for 20 h shows some corrosion and changes in the structure; all other samples remain largely unchanged, highlighting their good stability in aqueous solutions and towards illumination with UV light.

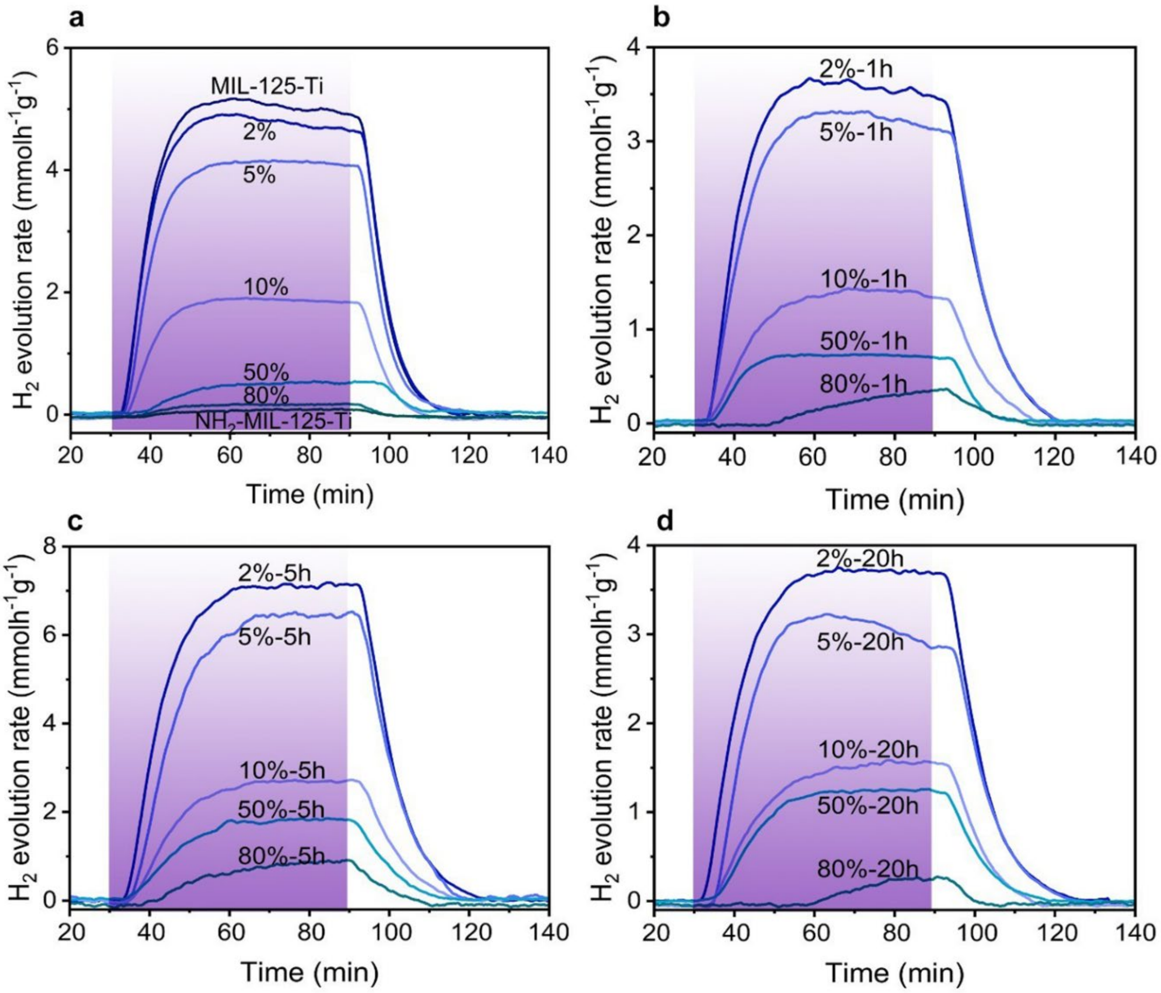

Supplementary Fig. 21 | HER performance of the MOFs. Temporal evolution of the H_2 evolution rates in mmol $h^{-1}g^{-1}$ during a photocatalytic experiment for (**a**) 2%, 5%, 10%, 50%, and 80%NH_2-MIL as-prepared mixed-ligand MOFs, (**b**) heat-treated MOFs for 1 h, (**c**) heat-treated MOFs for 5 h, (**d**) heat-treated MOFs for 20 h. The coloured area indicates UV illumination time (wavelength range: 280 nm – 400 nm).

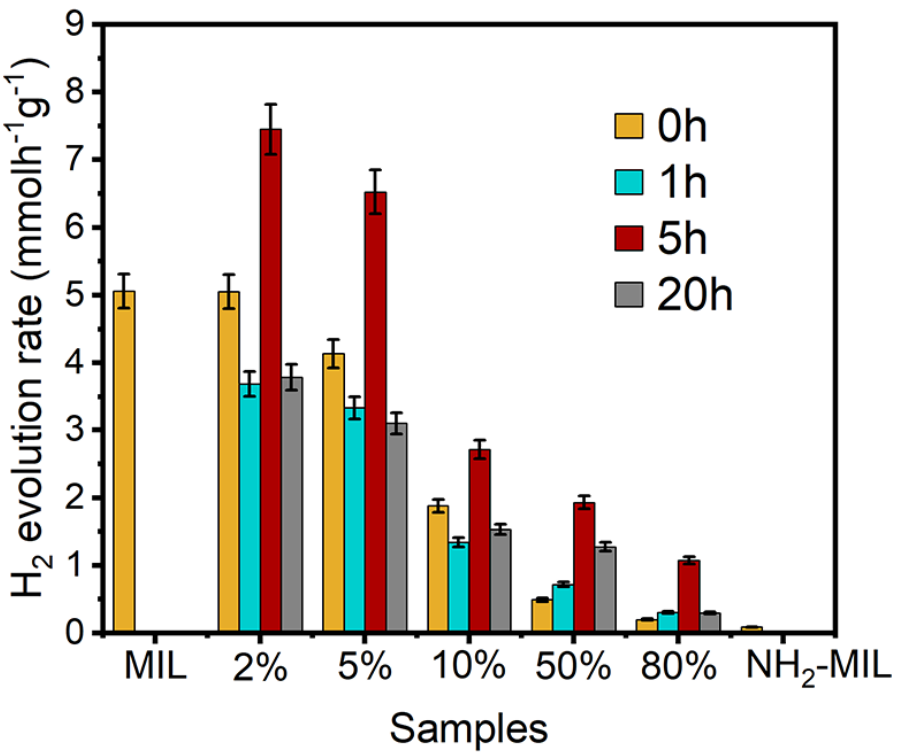

Supplementary Fig. 22 | Summary of HER activities of as-prepared and heat-treated samples. Note that the 2% and 5%NH_2-MIL samples outperform the MIL system after selective ligand removal. The rates generally decrease with increasing BDC-NH_2 content in both as-prepared and heat-treated samples, indicating that an increasing amount of residual linker is present after heat treatment with increasing original BDC-NH_2 content.

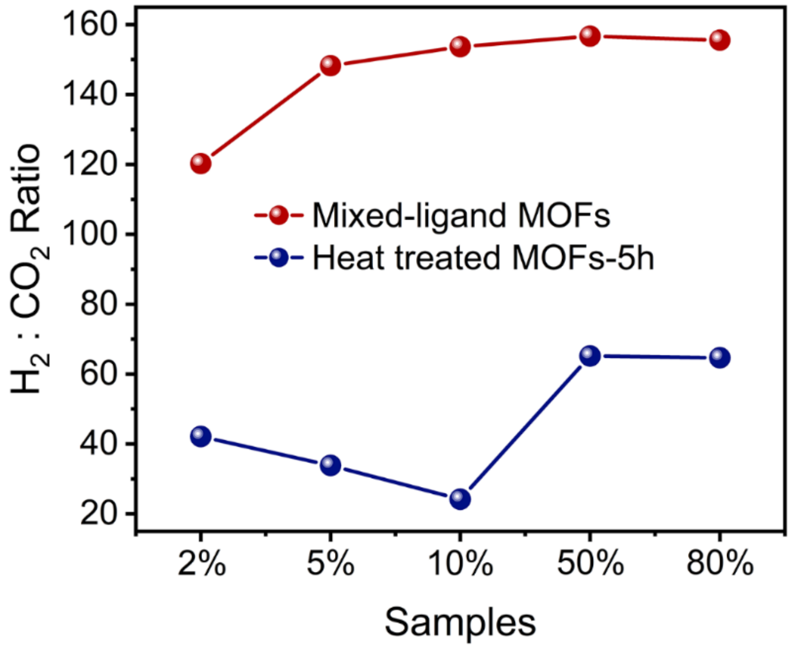

Supplementary Fig. 23 | The evolution rates of CO_2 (as the end product of total methanol mineralization) and the calculated H_2:CO_2 ratios. Since the ratios are well above a ratio of 3, as expected from methanol steam reforming, it can be deduced that most hydrogen comes from water rather than from methanol. The decrease in ratio after ligand removal suggests that hydrogen formation from methanol is favoured, likely due to the increase of new adsorption sites for methoxy. This indicates that methanol, which adsorbs on $Ti_8O_8(OH)_4$ clusters as methoxy groups and forms CO_2 via transfer of photoexcited holes[9], can access the interior of the frameworks more easily after selective ligand removal.

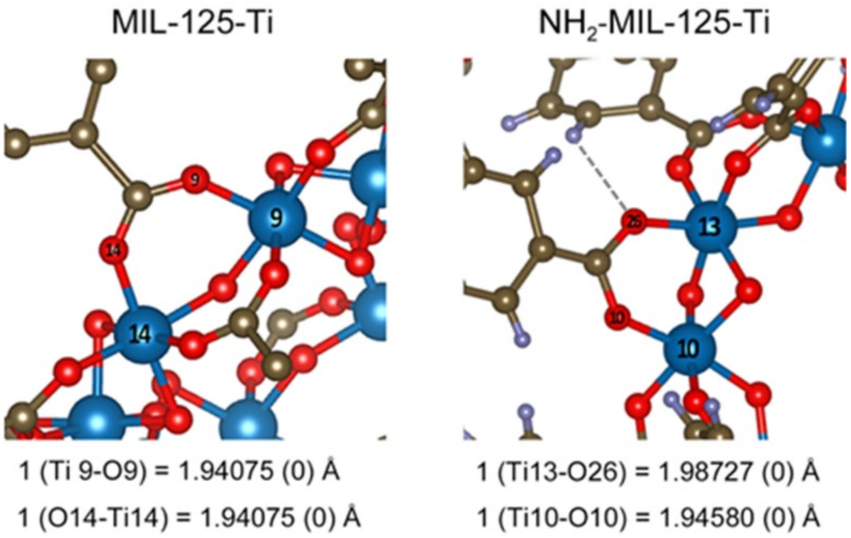

Supplementary Fig. 24 | The binding strength of the ligands with the Ti-SBU. The binding strength provides information about the ligand coordination to the SBU in MIL and NH_2-MIL. This value for the MIL sample is symmetric and yields a bond length of 1.941 Å. In contrast, the BDC-NH_2 asymmetry provides coordination with two O-Ti bond distances.

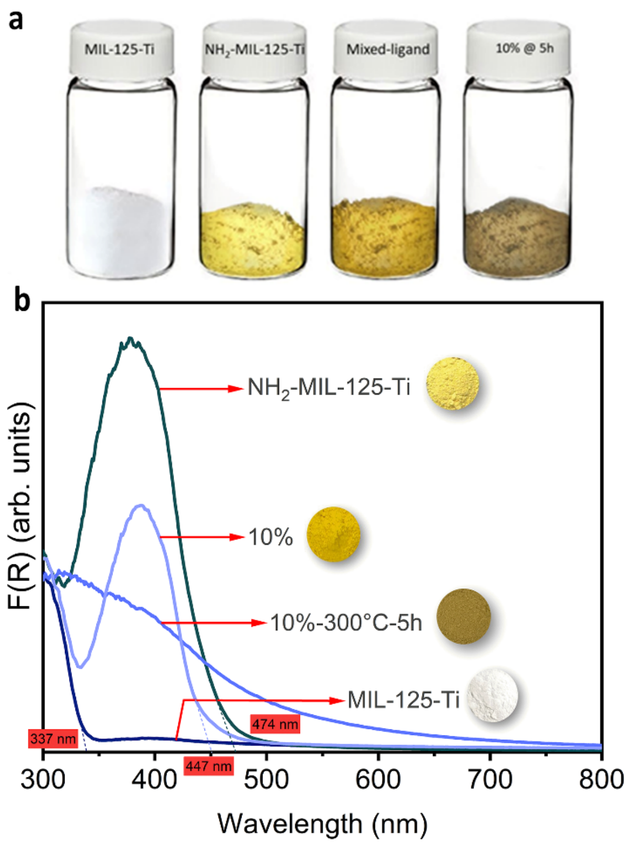

Supplementary Fig. 25 | Changes in electronic structure of the MOFs. (**a**) The colour of the as-prepared mixed-ligand MOFs and the corresponding heat-treated samples for 5 h for 10%NH_2-MIL MOF. The colour changes show white to light yellow colour with the increasing amount of BDC-NH_2. After selective ligand removal, the colour tends to be yellowish-brown depending on the ratio of BDC-NH_2 content, which is ascribed as partial oxidation of BDC-NH_2[18]. (**b**) Kubelka-Munk function spectra of MIL, NH_2-MIL, and 10%NH_2MIL mixed-ligand and heat-treated sample. The graph shows MIL starts to absorb the light from 337 nm while NH_2-MIL absorbs the light below 474 nm, in agreement with their colour and bandgap structure (3.60 eV and 2.68 eV, respectively). In NH_2-MIL, the absorption peak centred at 380 nm is thus attributed to the charge transfer from the –NH_2 group to the Ti-oxo cluster.

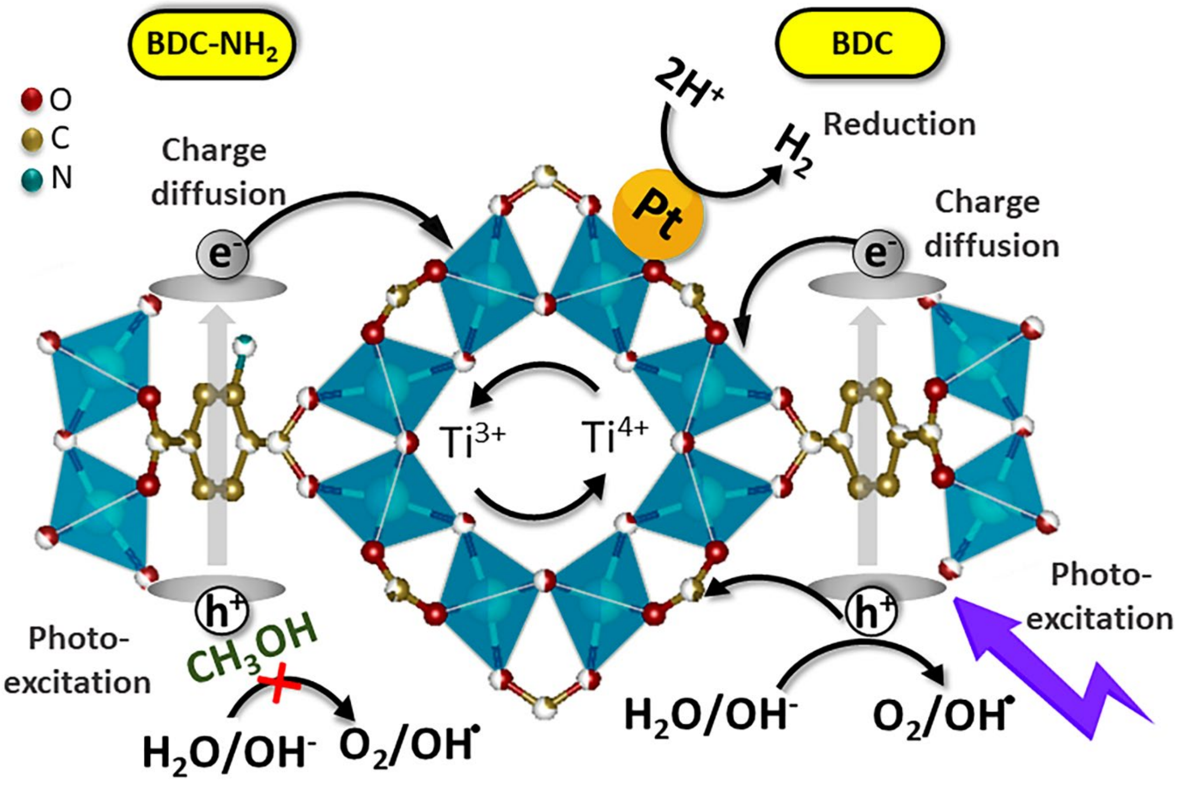

Supplementary Fig. 26 | Schematic representation of the proposed mechanism for photocatalytic hydrogen evolution in MIL and NH_2-MIL. Identifying adsorbed methoxy species as effective hole scavengers in NH_2-free MIL.

Supplementary Tables

Supplementary Table 1 | The synthesis details of the MOFs.

Samples		*Route I*			*Route II*		
$BDC\text{-}NH_2$ mol.%	0	2%	5%	10%	50%	80%	100%
BDC (mg)	1000	1000	1000	1000	249	99.8	0
$BDC\text{-}NH_2$ (mg)	0	22.2	57.2	120.8	271.7	434.7	568.1
TTIP (mL)	0.592	0.592	0.592	0.592	0.592	0.592	0.592
Solvent Volume (mL)	20	20	20	20	40	40	40
@ 150 °C	18 h	18 h	18 h	18 h	24 h	24 h	24 h

Supplementary Table 2 | IR characteristics bands observed in DRIFTS experiments.

**DRIFTS Peaks**		
Wave number (cm^{-1})	Vibration Type	Assignment
2945-2913		DMF
2851-2797		DMF
3663-3640		OH H-bonded
3705-3663		OH free
3625-3605		OH cleavage
3416-3335	stretching	NH_2 symetric
1265-1225		CN
1607-1575		COO- asymetric
1185-1134	stretching	C-O
1287-1273	stretching	C-O
1305-1287	stretching	C-O
1717-1695	stretching	C=O
1749-1717	stretching	C=O
1036-1005	bending	C-H Ring
762-741	bending	C-H Ring
782-762	bending	C-H Ring
974-959	bending	C-H Ring

Supplementary Table 3 | Particle thickness and diameter of the mixed-ligand MOFs and heat-treated MOFs for 5 h.

	Diameter (nm)		Thickness (nm)	
**Samples**	**Mixed-ligand**	**Heat-treated MOFs**	**Mixed-Ligand**	**Heat-treated MOFs**
2%	877.4	865.2	441.3	440.6
5%	801.8	799.6	383	375.1
10%	796.7	779.5	351.7	341.3
50%	849	845.4	382.7	380.3
80%	361.4	350.1	168.8	159.0

Supplementary Table 4 | The ratio of BDC-NH_2 in 2%, 5%, 10%, 50% and 80%NH_2-MIL, calculated by ^{1}HNMR.

Starting ratio of BDC-NH_2 (mol%)	2%	5%	10%	50%	80%
Actual ratio of BDC-NH_2 (mol%)	2%	5%	11%	47%	76%

Supplementary Table 5 | Summary of the S_A and pore volume of the mixed-ligand samples and heat-treated MOFs for 1, 5, and 20 h.

**Samples**	**Apparent specific surface area (m^2g^{-1})**	**NLDFT Micropore volume (cm^3g^{-1})**	**NLDFT Total pore volume (cm^3g^{-1})**
2% - 0h	1399	0.58	0.63
5% - 0h	1251	0.52	0.56
10% - 0h	1252	0.51	0.60
50% - 0h	1457	0.62	0.72
80% - 0h	1234	0.54	0.61
2% - 1h	1412	0.58	0.76
10% - 1h	1277	0.53	0.62
50% - 1h	1201	0.51	0.64
2% - 5h	917	0.39	0.62
5% - 5h	872	0.36	0.57
10% - 5h	748	0.30	0.56
50% - 5h	1151	0.49	0.61
80% - 5h	995	0.43	0.55
2% - 20h	1218	0.48	0.7
10% - 20h	197	0.05	0.23
50% - 20h	1010	0.41	0.56

Supplementary Table 6 | HER activities (mmol h^{-1} g^{-1}) calculated from the rates after 1 h of irradiation for the mixed-ligand and the heat-treated MOFs at 300 °C.

**Samples**	**Mixed-ligand MOFs**	**1h**	**5h**	**20h**
MIL	5.058	-	-	-
2%	5.053	3.689	7.450	3.785
5%	4.135	3.328	6.526	3.100
10%	1.879	1.342	2.714	1.533
50%	0.493	0.718	1.930	1.275
80%	0.200	0.307	1.076	0.297
NH_2-MIL	0.088	-	-	-

Supplementary Materials and Methods

Chemicals and reagents. Terephthalic acid (BDC, 98%, Merck), 2-Amino-1,4-benzenedicarboxylic acid (BDC-NH_2, >98.0%, TCI), Titanium (IV) isopropoxide ($C_{12}H_{28}O_4Ti$, TTIP, 97%, Sigma-Aldrich), N, N-dimethylformamide (DMF, 99.8% Extra Dry, ACROS), Methanol (MeOH, > 99.9% UHPLC, VWR), Hexachloroplatinic acid (H_2PtCl_6 8 wt. % in H_2O, Fluka/Sigma-Aldrich), Sulfuric acid (H_2SO_4, D_2, 99.00% in D_2O, Eurisotop), Dimethylsulfoxide D_6 (DMSO-d_6, 99.80%, Eurisotop).

Powder X-ray diffraction (PXRD). PXRD profiles were carried out on a PANalytical X'Pert Pro multi-purpose diffractometer (MPD) in Bragg Brentano geometry operating with a Cu anode at 45 kV, 40 mA, equipped with a BBHD Mirror and an X-Celerator multichannel detector. Samples were ground and placed as loose powders on silicon single crystal sample holders. The diffraction patterns were recorded at a 2θ angle of 5° and 50°; sample holders were rotated with 4 s per turn during the measurement. All measurements were conducted with Cu sealed tube $K_α$ and $K_ß$ radiation (2:1 ratio) with a wavelength of λ=1.54060 Å at a scan rate of 0.5° min^{-1}.

in situ X-ray diffraction (in situ XRD). in situ XRD was measured on a PANalytical, X´Pert Pro MPD diffractometer system, conducted with Cu-$K_{α1,2}$ radiation (λ= 1.54060 Å, 1.54439 Å) and equipped with an X-Celerator multichannel detector using Bragg Brentano geometry. The samples were heated with an Anton Paar HTK 1200 N oven under air (0.5 mL min^{-1}) up to 20 h with a ramping of 5 °C min^{-1}.

Scanning electron microscope (SEM). SEM images were taken using a FEI Quanta 250 FEG SEM. Small amounts of the samples were attached to the sample holder with conducting carbon tape. The images were recorded with an acceleration voltage of 10 kV and a beam current of 270 µA.

UV−Vis Diffuse Reflectance spectra. UV-Vis spectra were measured on a Jasco V-670 in diffuse reflectance mode with an Ulbricht-sphere. The incident light was in the range of 300 nm - 800 nm. $MgSO_4$ was used as a baseline, and the reflectance was converted according to Kubelka-Munk.

Argon physisorption isotherms. Ar physisorption isotherms were measured at 87 K on a Quantatec iQ2 instrument (Anton Paar, Boynton Beach, FL, USA) equipped with the "cryosync accessory." The samples were outgassed under vacuum at 150 °C for 12 h prior to measurement. The total pore volume of all samples was estimated from the amount of argon

adsorbed at P/P0 = 0.95, assuming that adsorption on the external surface was negligible compared to adsorption in the pores[10]. The apparent surface area was calculated using the Brunauer-Emmet-Teller (BET) equation and following the procedure recommended for microporous sorbents[11]. Relevant pore size distributions were calculated from the adsorption branch of the isotherms by applying the kernel of (metastable) nonlocal density functional theory (NLDFT) adsorption isotherms considering an amorphous SiO_2 surface and a cylindrical pore model. Micropore volume and total pore volume were also determined using the same kernel. The calculations were carried out using the ASiQwin 5.2 software provided by Quantachrome Instruments.

in situ Diffuse Reflectance for Infrared Fourier Transform Spectroscopy (DRIFTS). DRIFTS data were measured with an IR Tracer-100 (Shimadzu, Japan) under air atmosphere. Experiments were performed on powdered form of the samples, diluted by physically grinding of KBr as the reference. The instrument was equipped with a controlled heating device.

Thermogravimetric analysis (TGA). TGA measurements were conducted on a PerkinElmer Thermogravimetric analyser 8000 (Waltham, USA), using an aluminium oxide (Al_2O_3) crucible. Air was used as treatment gas with a ramp rate of 10 °C min^{-1}.

Differential scanning calorimetry (DSC). DSC was performed at a heating rate of 10 °C min^{-1} under a flowing air atmosphere with 20 mL min^{-1} flow rate over a temperature range of 50 to 600 °C on a Mettler Toledo (DSC 823) using aluminium pans with pierced lids.

Nuclear Magnetic Resonance spectroscopy (^{1}HNMR). ^{1}H spectra were recorded on a Bruker ADVANCE 250 (250.13 MHz) equipped with a 5 mm inverse-broad probe head and z-gradient unit. DMSO-*d6*: δ = 2.50 ppm was used as an internal reference. ^{1}HNMR spectra were taken of mixed-ligand as-prepared and heat-treated 10%NH_2-MIL at 300 °C for 1, 5 and 20 h. Due to low solubility, 2 mg of the samples were digested with 10 drops of concentrated H_2SO_4 and sonicated until the sample was well dispersed in the acid. Then, 0.5 mL of DMSO-d_6 was added to the solution[12].

X-ray photoelectron spectroscopy (XPS). XPS spectra were acquired in a stainless-steel UHV chamber (base pressure < 3×10^{-10} mbar) equipped with a Specs XR50© high intensity non-monochromatic Al/Mg dual anode and a Phoibos 100© hemispherical electron energy analyzer with multichannel plate detector[13]. Powdered samples were deposited on conducting adhesive Cu tape (SPI Supplies©), with XPS spectra (Al K_α radiation of 1486.61 eV and photoelectron emission angle of 0°) acquired at room temperature. For data evaluation, CasaXPS was employed. XPS peaks of Ti 2p were referenced to the C 1s signal at 284.6 eV

and were fitted after Shirley background subtraction utilizing asymmetric Lorentzian (LA) functions.

Transmission electron microscope (TEM). The electron microscopy studies were conducted on FEI Tecnai F20TEM (Thermo Fischer Scientific, a facility at USTEM (University Service Centre for Transmission Electron Microscopy) at TU Wien with an acceleration voltage of 200 kV. The powder samples were dispersed in EtOH by sonicating them in an ultra-sonic bath for 5 min. The dispersed solution was then drop-casted onto meshed copper grids, coated with a holey carbon film, and were left to dry at ambient atmosphere.

Raman Spectroscopy. Raman spectroscopic measurements employed Horiba Jobin-Yvon LabRAM 800HR and were carried out using 532 nm wavelength green laser. The Raman measurements were performed by placing powder samples on a glass slide. For initial calibration studies silicon reference sample was used to align the silicon Raman band at 520cm^{-1}.

Density-functional theory (DFT). All simulations have been performed by using the Vienna ab initio simulation package (VASP 5.4.1)[14,15] under spin-polarized density functional theory (DFT)[16,17]. We performed the simulations using the Perdew-Burke-Ernzerhof (PBE) exchange-correlation (XC) functional[18] with a Hubbard-like U correction term (U = 3.5 eV) to account for the on-site Coulombic interactions of the titanium d electrons, as proposed by Dudarev et al.[19–21]. Projected augmented waves (PAWs)[22,23] represented the inner core electrons and nuclei on each atom. For Ti, O, C, N atoms, the appropriate PAW potentials replaced the [Ar], [He], [He], and [He] inner shell electrons, respectively. We used an energy cut off of 520 eV to model the electronic wave functions, and all simulations have been performed at the Γ-point only inside the Brillouin zone. For all the simulations, we used electronic convergence with a tolerance of 10^{-4} eV, and ionic relaxations took place with a conjugate gradient algorithm[24], and forces were converged to a tolerance of 0.03 eV/Å. Ionic relaxation was done with the Gaussian smearing method using a smearing width of 0.05 eV. Vibrational frequencies were determined by using the finite differences method implemented in VASP, that is, by displacing a selected number of atoms in the direction of each Cartesian coordinate to determine the Hessian matrix.

Photocatalysis experiment. Photocatalytic evolution tests were carried out in a custom-made flow reactor. The temperature was kept constant at 15 °C by using water cooling. The reactor was constantly purged with argon during the reaction, which was used as a carrier gas. The argon gas flow was controlled via mass flow controller (mL min^{-1}) (Q-flow 140 series, MCC-Instruments). The produced gases were carried with the Ar flow from the reactor to the

detector. The selected amount of powdered photocatalyst (standard: 0.01 g) was dispersed in a 40 mL aqueous methanol solution (50 vol%) in each experiment. In the next step, a certain amount of an aqueous H_2PtCl_6 solution for in situ photodeposition (chosen to correspond to the desired amount 0.5 wt% of Pt NPs as co-catalyst) was added to the solution before closing the reactor. After equilibration in the dark, the UV light was turned on for 1 h. The chemical composition of the reaction volume was detected during the whole experiment utilizing an Emerson detector that contains infrared-, piezo- and thermal conductivity detectors to quantify on-line up to four gases simultaneously. After the UV was turned off, the system was allowed to equilibrate in the dark for about 1 h. Recovering the catalyst was done by centrifuging the solution after the experiment.

Supplementary Notes | Synthesis

Synthesis of MIL-125-Ti MOF. In a typical experiment, BDC (1 g, 6 mmol) was dissolved in a solution of DMF and methanol (20 mL, $V_{DMF}/V_{methanol}$ = 9:1). Then TTIP (0.592 mL, 2.0 mmol) was added to the BDC solution. After stirring by ultrasonic for 5 minutes, the mixture was transferred to a 45 mL Teflon-lined steel autoclave and placed in an oven at 150 °C for 18 h under static conditions. After cooling, the white solid product was washed three times with DMF and twice with methanol and then centrifuged. Samples were dried for 1 day in a vacuum oven at 150 °C to remove any free solvents.

Synthesis of NH_2-MIL-125-Ti MOF. For the synthesis of NH_2-containing MOF, denoted as NH_2-MIL, 2-aminoterephthalic acid (BDC-NH_2) (568.1 mg, 3 mmol) was added to 40 mL of the DMF/methanol solution ($V_{DMF}/V_{methanol}$ = 9:1). Then TTIP (0.592 mL, 2.0 mmol) was added to the solution. After stirring for 5 minutes, the solution was transferred to a 100 mL Teflon-lined steel autoclave at 150 °C for 24 h. After cooling, the yellow solid product was washed three times with DMF and twice with methanol and then centrifuged. Samples were dried for 1 day in a vacuum oven at 150 °C.

Synthesis of mixed-ligand MOFs. For the mixed-ligand MOFs the content of BDC-NH_2 was denoted as xNH_2-MIL when x= 2, 5, 10, 50, 80%. TTIP (0.592 mL), BDC (1000 mg for 2, 5, 10% and 249 and 99.8 mg for 50, 80% respectively), BDC-NH_2 (22.2, 57.2, 120.8, 271.7, 434.7 mg, respectively), were added to a solution of DMF and methanol (20 mL for 2, 5, 10% and 40 mL for 50, 80%, $V_{DMF}/V_{methanol}$ = 9:1) and poured in a 100 mL Teflon-lined steel autoclave. The mixture was heated at 150 °C for 18 h (2, 5, and 10%) and 24 h (50 and 80%). As the non-dominant linker dispersed in the matrix of dominant linker[25], we used 2 different routes to synthesize the mixed-ligand MOFs: route 1 is based on adding BDC-NH_2 to the MIL-125-Ti precursor and the route 2 added BDC ligand to NH_2-MIL precursor. The samples with 2, 5, and 10% of NH_2 content were synthesized by route 1, and route 2 was applied to prepare 50 and 80%. After cooling to room temperature, the resulting powder was washed with DMF three times and twice with methanol to remove the unreacted ligand, separated by centrifugation, and dried under vacuum at 150 °C for 1 day.

Heat-treated MOFs. Two protocols followed: 1) The samples were heated in air with a constant ramp (10 °C min^{-1}) up to 550 °C. 2) The samples were heated to 300 °C with a constant ramp (10 °C min^{-1}) and kept at that temperature for durations up to 20 hours. In both cases, the samples were allowed to cool down to room temperature.

Preparation of Pt co-catalysts solution. A $4.1 \cdot 10^{-3}$ M stock solution was made from 0.5 mL of the aqueous 8 wt% H_2PtCl_6 (M = 409.81 g mol^{-1}) solution that was diluted with deionized water to 25 mL. With a catalyst amount of 10 mg, the Pt loading was calculated to be 0.5 wt%. The hydrogen production from acid reduction corresponds to 62.51 µL of H_2PtCl_6 stock solution; this amount was compensated for in the photocatalytic tests.

Supplementary Notes | DFT calculations

Input parameters (INCAR tags, VASP format) for the Simulation:

ICHARG = 2; ISTART = 0; ISYM = 2; NPAR = 8; MAGMOM = Ti*3 O*0 C*0 N*0 H*0; ALGO = Normal; PREC = Accurate; EDIFF = 1e-4; EDIFFG = -0.03; ISMEAR = 0; SIGMA = 0.05; NSW = 50; NELM = 60; ENCUT = 520; LREAL = Auto; ISPIN = 2; NSW = 50; IBRION = 2; POTIM = 0.5; ISIF = 2; LDAUU = 3.5 (for Ti); LWAVE = .TRUE.; LCHARG = .TRUE.

Input parameters (INCAR tags, VASP format) for the Vibrational frequencies:

NSW = 1; IBRION = 5; POTIM = 0.02; NFREE = 2; NWRITE = 3

Simulated structures with total energies:

Simulation before cleavage (NH_2-MIL-125-Ti): Etot = - 1833.6006 eV simulation after cleavage (NH_2-MIL-125-Ti): Etot = - 1833.7629 eV

Supplementary References

1. Abdelhameed, R. M., Simões, M. M. Q., Silva, A. M. S. & Rocha, J. Enhanced Photocatalytic Activity of MIL-125 by Post-Synthetic Modification with CrIII and Ag Nanoparticles. *Chem. – Eur. J.* **21**, 11072–11081 (2015).
2. Weirich, T. E., Winterer, M., Seifried, S. & Mayer, J. Structure of nanocrystalline anatase solved and refined from electron powder data. *Acta Crystallogr. A* **58**, 308–315 (2002).
3. Zhang, W. F., He, Y. L., Zhang, M. S., Yin, Z. & Chen, Q. Raman scattering study on anatase TiO2nanocrystals. *J. Phys. Appl. Phys.* **33**, 912–916 (2000).
4. Hlophe, P. V. & Dlamini, L. N. Synthesis of a semi-conductor-like MOF with black phosphorous as a composite for visible light-driven photocatalysis. *RSC Adv.* **9**, 37321–37330 (2019).
5. Nanoengineering with residual catalyst from CNT templates | Elsevier Enhanced Reader. https://reader.elsevier.com/reader/sd/pii/S1359645410002533?token=7EB3893B2A3BCC89557C4DE78A51BB36591CDF24052DC6341E109183797C66233C804B7147AD5CD7020D8DE6BBDFAD8F&originRegion=eu-west-1&originCreation=20211119174449 doi:10.1016/j.actamat.2010.04.037.
6. MacGillivray, L. R., Groeneman, R. H. & Atwood, J. L. Design and Self-Assembly of Cavity-Containing Rectangular Grids. *J. Am. Chem. Soc.* **120**, 2676–2677 (1998).
7. Zhou, F., Zhou, J., Gao, X., Kong, C. & Chen, L. Facile synthesis of MOFs with uncoordinated carboxyl groups for selective CO2 capture via postsynthetic covalent modification. *RSC Adv.* **7**, 3713–3719 (2017).
8. Haselmann, G. M. *et al.* In Situ Pt Photodeposition and Methanol Photooxidation on Pt/TiO2: Pt-Loading-Dependent Photocatalytic Reaction Pathways Studied by Liquid-Phase Infrared Spectroscopy. *ACS Catal.* **10**, 2964–2977 (2020).
9. Wang, J. *et al.* Ti-based MOFs: New insights on the impact of ligand composition and hole scavengers on stability, charge separation and photocatalytic hydrogen evolution. *Appl. Catal. B Environ.* **283**, 119626 (2021).
10. Aharen, T. *et al.* Novel Co-based metal–organic frameworks and their magnetic properties using asymmetrically binding 4-(4′-carboxyphenyl)-1,2,4-triazole. *Dalton Trans.* **42**, 7795 (2013).
11. Sánchez-Varretti, F. O., García, G. D., Ramirez-Pastor, A. J. & Romá, F. A simple model for studying multilayer adsorption of noninteracting polyatomic species on homogeneous and heterogeneous surfaces. *J. Chem. Phys.* **130**, 194711 (2009).
12. Howarth, A. J. *et al.* Best Practices for the Synthesis, Activation, and Characterization of Metal–Organic Frameworks. *Chem. Mater.* **29**, 26–39 (2017).
13. Haunold, T. & Rupprechter, G. LiOx-modification of Ni and Co3O4 surfaces: An XPS, LEIS and LEED study. *Surf. Sci.* **713**, 121915 (2021).
14. Kresse, null & Hafner, null. Ab initio molecular dynamics for liquid metals. *Phys. Rev. B Condens. Matter* **47**, 558–561 (1993).
15. Kresse, G. & Furthmüller, J. Efficiency of ab-initio total energy calculations for metals and semiconductors using a plane-wave basis set. *Comput. Mater. Sci.* **6**, 15–50 (1996).
16. Hohenberg, P. & Kohn, W. Inhomogeneous Electron Gas. *Phys. Rev.* **136**, B864–B871 (1964).
17. Kohn, W. & Sham, L. J. Self-Consistent Equations Including Exchange and Correlation Effects. *Phys. Rev.* **140**, A1133–A1138 (1965).
18. Perdew, J. P., Burke, K. & Ernzerhof, M. Generalized Gradient Approximation Made Simple. *Phys. Rev. Lett.* **77**, 3865–3868 (1996).

19. Dudarev, S. L., Botton, G. A., Savrasov, S. Y., Humphreys, C. J. & Sutton, A. P. Electron-energy-loss spectra and the structural stability of nickel oxide: An LSDA+U study. *Phys. Rev. B* **57**, 1505–1509 (1998).
20. Aschauer, U., Chen, J. & Selloni, A. Peroxide and superoxide states of adsorbed O2 on anatase TiO2 (101) with subsurface defects. *Phys. Chem. Chem. Phys.* **12**, 12956–12960 (2010).
21. Langhammer, D., Kullgren, J. & Österlund, L. Photoinduced Adsorption and Oxidation of SO2 on Anatase TiO2(101). *J. Am. Chem. Soc.* **142**, 21767–21774 (2020).
22. Kresse, G. & Joubert, D. From ultrasoft pseudopotentials to the projector augmented-wave method. *Phys. Rev. B* **59**, 1758–1775 (1999).
23. Blöchl, P. E. Projector augmented-wave method. *Phys. Rev. B* **50**, 17953–17979 (1994).
24. Introduction. in *Numerical Optimization* (eds. Nocedal, J. & Wright, S. J.) 1–9 (Springer, 2006). doi:10.1007/978-0-387-40065-5_1.
25. Feng, L., Wang, K.-Y., Day, G. S. & Zhou, H.-C. The chemistry of multi-component and hierarchical framework compounds. *Chem. Soc. Rev.* **48**, 4823–4853 (2019).
